# Van der Waals Epitaxy of III-Nitrides and Its Applications

**DOI:** 10.3390/ma13173835

**Published:** 2020-08-31

**Authors:** Qi Chen, Yue Yin, Fang Ren, Meng Liang, Xiaoyan Yi, Zhiqiang Liu

**Affiliations:** 1 Research and Development Center for Solid State Lighting, Institute of Semiconductors, Chinese Academy of Sciences, Beijing 100083, China; chenq@ncepu.edu.cn (Q.C.); yinyue@semi.ac.cn (Y.Y.); rf@semi.ac.cn (F.R.); liangmeng@semi.ac.cn (M.L.); 2 Center of Materials Science and Optoelectronics Engineering, University of Chinese Academy of Sciences, Beijing 100049, China; 3 Beijing Engineering Research Center for the 3rd Generation Semiconductor Materials and Application, Beijing 100083, China

**Keywords:** graphene, van der Waals epitaxy, III-nitrides

## Abstract

III-nitride semiconductors have wide bandgap and high carrier mobility, making them suitable candidates for light-emitting diodes (LEDs), laser diodes (LDs), high electron mobility transistors (HEMTs) and other optoelectronics. Compared with conventional epitaxy technique, van der Waals epitaxy (vdWE) has been proven to be a useful route to relax the requirements of lattice mismatch and thermal mismatch between the nitride epilayers and the substrates. By using vdWE, the stress in the epilayer can be sufficiently relaxed, and the epilayer can be easily exfoliated and transferred, which provides opportunities for novel device design and fabrication. In this paper, we review and discuss the important progress on the researches of nitrides vdWE. The potential applications of nitride vdWE are also prospected.

## 1. Introduction

Inorganic semiconductors possess many advantages over organic semiconductors, including high carrier mobility, long-term stability, and reliability. Compared with elemental semiconductors (e.g., Si and Ge), III-nitrides semiconductors, such as AlN and GaN, have higher carrier mobility and radiation recombination rate, which makes III-nitrides perfect candidates for light-emitting diodes (LEDs), lasers (LDs), and high electron mobility transistors (HEMTs) [1]. Additionally, III-nitrides have broad bandgap coverage, therefore, adjusting the composition of the AlGaInN alloy can realize the bandgap continuously tunable from 0.64 eV (InN) to 6.2 eV (AlN), covering the spectrum from infrared to ultraviolet [2]. This makes III-nitrides have excellent application prospects in the fields of full-color display and photoelectric detection. However, the disadvantages of the traditional covalent epitaxy limit the application scenarios of III-nitrides. For example, due to the lattice mismatch and thermal mismatch between the substrate and epilayer, the stress in the epilayer cannot be sufficiently released, which introduces lots of defects in the initial stage of epigrowth. This is not expected when preparing high-performance films [3]. Besides, the epilayer interacts with the substrate through chemical bonds. This strong interaction is not conducive for the exfoliation and transfer of the epilayer and limits the development of III-nitride-based flexible and wearable electronic devices. The small size and low thermal conductivity characteristics of single crystalline substrates also restrict large-scale III-nitride film preparation as well as III-nitride-based high-power device fabrication [4,5] Fortunately, the van der Waals epitaxy can solve the problems mentioned above.

Since Koma et al. first demonstrated the Se/Te and NbSe_2_/MoS_2_ structure in 1984 [6], van der Waals epitaxy has been proved to be a promising heteroepitaxy approach. Van der Waals epitaxy is also a heteroepitaxy method for film growth, the difference is that the epilayer and the substrate are combined by the weak van der Waals force, rather than the strong chemical bond. The free-of-dangling bonds characteristic of 2D materials makes them suitable for being the interlayer of van der Waals epitaxy. Introducing 2D materials (single- or multilayer) into the III-nitride van der Waals epitaxy process can bring many novel features. Since the epilayer and the substrate interact with each other through weak van der Waals force, the lattice arrangement of the epilayer in the initial growth stage is not completely consistent with that of the substrate but maintains its own inherent lattice constant. That is to say, the residual stress in the as-grown epilayer is negligible, while the epilayer orientation can be still influenced by the substrate at the same time. Choosing van der Waals epitaxy as a heteroepitaxy method has many advantages, including releasing the lattice mismatch and thermal mismatch requirements between the epilayer and the substrate, easier exfoliating and transferring the epilayer from the substrate, and substrate reusing. These can contribute to novel device fabrication and cost reduction [7,8,9].

Although van der Waals epitaxy has been proved to be a competitive method for the III-nitride film epitaxy (Table 1), there are still some challenges worth exploring. In this paper, we will discuss the published research about III-nitride van der Waals epitaxy and deliver the prospect and future development of it. The paper is divided into four parts. The first part introduces the 2D materials used in van der Waals epitaxy, including its types (this paper mainly focuses on graphene), preparation methods, advantages, and disadvantages. The nucleation growth and lattice orientation mechanism of III-nitride van der Waals epilayer are described in the second part, the cases of optoelectronic devices fabricated by van der Waals epitaxy are delivered in the following part. Finally, in the fourth part, both the summary and the prospect of III-nitride van der Waals epitaxy are given.

## 2. 2D Materials and van der Waals Epitaxy

Since Koma et al. published their research in 1984, many researchers have been trying to replace the strong chemical bond by the weak van der Waals force for film epitaxy and have succeeded in some compounds. Then, Geim et al. fabricated single-layer graphene through mechanical exfoliation method in 2004 [16], the existence of 2D materials was demonstrated and soon triggered researchers’ interest.

Basically, 2D material is a planar material and its in-plane atoms are generally arranged in a honeycomb structure. Up to now, different kinds of 2D materials have been discovered, such as graphene, hexagonal boron nitride (h-BN), and transition metal dichalcogenides (TMDCs). Comparing with 3D bulk materials, 2D materials have many unique properties. The in-plane atoms of 2D materials interact with each other through strong chemical bonds, while the interaction between adjacent layers is weak van der Waals force, which is beneficial for epilayer peeling-off. The free-of-dangling bonds characteristic of 2D materials ensures high-quality film preparation despite the limitation of lattice mismatch and thermal mismatch [8,17]. However, it also decreases the nucleation density on the 2D material [9], which may affect the continuity and smoothness of the epilayer.

Graphene is the first 2D material discovered by the scientific community and often acts as a buffer layer for the III-nitride van der Waals epitaxy process. Using graphene as the III-nitride van der Waals epitaxy buffer layer has many incomparable advantages, one of which is that the preparation methods of graphene are diverse [18,19,20]. The methods of graphene preparation can be roughly divided into two categories. One is to directly exfoliate the graphene from the graphite through external physical or chemical force. For example, the mechanical exfoliation method refers to directly destroying the van der Waals force between the graphite layers by the external force to obtain graphene [16]. The oxidation–reduction method is to separate graphite layers by oxidation, and then reduce the obtained graphene oxide to graphene [21]. The liquid-phase exfoliation method is to disperse the graphite in a solvent followed by graphene layer exfoliation with the assistance of ultrasound and finally separate exfoliated from unexfoliated flakes via ultracentrifugation. The other is to synthesis graphene directly, such as the chemical vapor deposition (CVD) method and epitaxy method. CVD method means using carbon precursor and metal catalyst (usually Ni or Cu) to realize directly synthesizing graphene on a metal foil. In epitaxy method, the SiC substrate is heated under ultrahigh vacuum condition, and then the C atoms reconstruct to the graphene structure while the Si atoms sublimate away [22,23].

Different graphene preparation methods have their own advantages and disadvantages. As for III-nitride van der Waals epitaxy, complete and continuous graphene is needed. Among the graphene preparation methods, the graphene prepared by the epitaxy method on a SiC substrate is known to achieve higher quality. In 2017, Yu Xu et al. prepared multilayer graphene by sublimating Si on the SiC substrate and successfully carried out the AlN epitaxy [24]. Besides, the epitaxial graphene formed by sublimation of silicon from the surface of SiC (0001) can be converted to quasi-freestanding epitaxial graphene (QFEG) via hydrogenation, which can be used for 2D GaN fabrication. Al Balushi et al. realized 2D GaN growth between bilayer graphene and SiC (0001) in 2016. Based on their DFT calculation result, this can be expected to open up new avenues of research in electronic and optoelectronic devices composed of 2D III-nitride atomic layers [25]. Graphene prepared through the CVD method also performs well in the van der Waals epitaxy process on both amorphous and crystalline substrates. Hong et al. and Qi et al. transferred CVD prepared graphene onto amorphous substrate and sapphire substrate as the buffer layer before GaN epitaxy, respectively [26,27]. Except for transferring the CVD graphene grown on Cu or Ni foil to the target substrate, graphene can be directly grown on the target substrate. In 2018, Chen et al. directly prepared graphene on the sapphire substrate through catalyst-free atmospheric pressure chemical vapor deposition (APCVD) [28]. Directly growing the graphene on the target substrate avoids the damage caused by the tedious exfoliate and transfer process, which effectively improves the quality of graphene [29,30].

In addition to diverse preparation methods, many inherent physical and chemical properties of graphene also determine its suitability for III-nitride-based optoelectronic devices. MOCVD is commonly used for III-nitride epigrowth because it can control the composition, thickness, and interface of the epilayer by adjusting the flow rate, species, and flux time of precursors, as confirmed by a DFT calculation done by A. Kakanakova-Georgieva et al. [31]. Graphene can meet the temperature requirements of III-nitride MOCVD growth (up to 1000 °C) due to its high melting point and chemical inertness. In recent years, many researchers have successfully achieved AlN and GaN van der Waals epigrowth by using graphene as a buffer layer. Besides, graphene has excellent electrical and thermal conductivity as well as optical transparency [32,33,34], making it qualified for being the device’s transparent conductive electrode without exfoliating from the epitaxy structure. Chung et al. transferred the graphene as the transparent electrode along with the GaN-based LED structure to different substrates in 2010 [9]. In 2015, Heilmann et al. also pointed out that GaN nanorods grown on insulating substrates like sapphire can be contacted from underneath by inserting a graphene layer at the interface with the substrate before their actual growth [35].

Moreover, metal atoms, such as Ga and Al, can migrate quickly on the graphene surface due to their low migration barrier on graphene. The quick migration of atoms is beneficial for nuclei island coalescence, which can contribute to the continuous film formation and epitaxy time reduction [36,37]. As pointed out by Chen et al. in 2019, a graphene interlayer can contribute to quick mirror-smooth AlN epilayer formation, the epitaxial time is reduced by 50% compared with the traditional epitaxy method [14]. Besides, the weak interaction between the graphene layers is conducive for the epilayer exfoliation, which can facilitate the subsequent transfer of III-nitride film to the target substrate and thus broaden the application scenarios of III-nitrides. For example, the high electrical and thermal conductivity of metal substrates is beneficial to the preparation of high-power electronic devices. The optical transparency of the glass substrates as well as the excellent flexibility of plastic substrates can contribute to full-color display and flexible device fabrication, respectively. In 2010, Chung et al. demonstrated that the GaN-based LEDs fabricated by the van der Waals epitaxy method can still maintain an identical brightness after transferred to the metal, glass, and plastic substrate [9]. Subsequently, in 2014, they achieved flexible GaN-based LED fabrication by transferring the LED structure from the graphene/amorphous substrate to the PI substrate [38].

The free-of-dangling bonds characteristic of graphene is crucial for the van der Waals epitaxy, but it also causes problems. Due to the low chemical reactivity of graphene surface, III-nitrides only tend to nucleate at ridges and steps of graphene, which will result in low nucleation density and eventually affect the film morphology. Therefore, the graphene treatment procedure is generally needed for increasing its surface chemical reactivity. Plasma treatment, usually N_2_ or O_2_, is one of the feasible methods [39]. Typically, the purpose of plasma treatment is to increase the surface defect density of graphene, namely, to improve the chemical reactivity of the graphene surface. Chen et al. achieved favorable AlN van der Waals epitaxy growth on N_2_ plasma-treated graphene in 2019 [14]. They pointed out that the defect density in graphene was increased from n_D_ = 2.13 × 10^11^ cm^−2^ to n_D_ = 3.23 × 10^11^ cm^−2^ after the N_2_ plasma treatment and the nitrogen incorporation formed new N-sp^3^ C bonds. DFT calculation verified that the adsorption energy of Al atoms to pyrrolic N in plasma-treated graphene was greatly increased to 5.9–8.6 eV, compared with the adsorption energy of Al atoms to graphene, 1.1 eV. Thus, the reactivity of graphene for AlN nucleation was enhanced. However, some studies have demonstrated that an ideal nucleation density of III-nitrides can be obtained by taking advantage of graphene-inherent defects, such as ridges and steps. In 2012, Choi et al. enhanced graphene surface chemical reactivity by directly reducing the size of graphene grains, thus sufficient nucleation density was obtained owing to the increased number of graphene ridges [40]. Some other studies indicate that the damages caused by the graphene transfer process can also act as III-nitride nucleation sites. Li et al. successfully carried out AlN film fabrication through van der Waals epitaxy on untreated wet transferred multilayer graphene in 2017 [41]. Subsequently, in 2019, Wang et al. achieved an AlGaN nanostructure growth by introducing an untreated transferred graphene layer on the amorphous SiO_2_/Si substrate [42].

Except for graphene, transition metal dichalcogenides are also used as the buffer layer in van der Waals epitaxy process of III-nitrides. Using two-dimensional WS_2_ and MoS_2_ as III-nitride epitaxy buffer layer may greatly reduce the dislocation density in the epilayer because the lattice mismatch between them and GaN is only 1.0% and 0.8%, respectively [43]. Yin et al. obtained smooth AlN film with the introduction of the WS_2_ buffer layer and subsequently implemented the full-structure growth of DUV-LED in 2018 [44]. In 2017, Zhao et al. achieved InGaN/GaN nanowire LEDs fabrication on sulfided Mo substrates [45]. Besides, van der Waals stacked hybrid solid materials may also be used for III-nitride van der Waals epitaxy for its interesting electrical, mechanical, and optical properties as demonstrated by Gao et al. in 2012. They performed a DFT calculation to determine the electronic and structural properties of solids consisting of randomly stacked layers of graphene and h-BN (h-BN/G solids) and found that the stacked h-BN/G solids’ properties were distinctly different from their starting parent layers due to a large number of interfaces between electronically dissimilar, flat, atomic layers bound through weak van der Waals forces [46].

As mentioned above, 2D materials, especially the graphene, play an important role during the van der Waals epitaxy process of III-nitrides. The detailed van der Waals epitaxy process of III-nitrides will be discussed in the following part.

## 3. Van der Waals Epitaxy of III-Nitrides on Graphene

Similar to the traditional epitaxy, the van der Waals epitaxy process includes the precursors adsorption-nucleation, nucleus growth, and nuclei island coalescence procedures. However, it is worth noting that the impact of the 2D material on van der Waals epitaxy cannot be ignored. On the one hand, the potential of the substrate can be partly screened by the two-dimensional buffer layer, which may influence the crystal orientation and the lattice constant. On the other hand, the inherent chemical and physical properties of the 2D material also have a certain effect during the nucleation-growth process of the epilayer. Nowadays, many studies have confirmed the feasibility of III-nitrides van der Waals epitaxy on graphene, and the following paragraphs will discuss it based on the inherent physical and chemical properties of graphene.

As mentioned above, graphene has the following significant characteristics when being the buffer layer of van der Waals epitaxy. First, the surface of graphene lacks dangling bonds, so generally, additional treatment is required for the ideal nucleation density. Second, metal atoms, such as Al and Ga, can migrate rapidly on the graphene surface, which is beneficial for the coalescence process. Third, the weak interaction between the adjacent graphene layers allows the epilayer to be exfoliated and transferred easily.

The methods for increasing the chemical reactivity of the graphene surface are discussed in the previous section. This section will focus on the III-nitride nucleation-growth process on graphene. The concepts of adsorption energy and migration barrier are needed here. When the moving molecules slow down and eventually adsorb on the surface of the medium, an energy release is generated. The released energy is called adsorption energy. For the same medium, the atom with higher adsorption energy is more likely to exist stable on its surface [37]. As for the van der Waals epitaxy process on graphene, the ratio of adsorption energy to bulk cohesive energy (i.e., the energy released when the atoms combine into the bulk material) (E_b_/E_c_) should be concerned. For III-nitrides, a higher E_b_/E_c_ ratio means easier to adsorb and nuclei on the graphene and exhibit a 2D growth mode [47]. Besides, the migration of atoms on graphene should also be considered. Due to the low surface free energy of graphene, the migration barrier for the atoms on it is much lower than that on 3D bulk materials. That is to say, the diffusion length and migration rate of the atoms will become lager on graphene. Overall, it is necessary to analyze the adsorption energy as well as the migration barrier on graphene for a certain material before the van der Waals epitaxy. The atom’s adsorption energy on graphene should not be so low that it affects the adsorption stability, and the migration barrier should not be too high otherwise, it may induce the 3D island growth mode.

**Figure 1:**
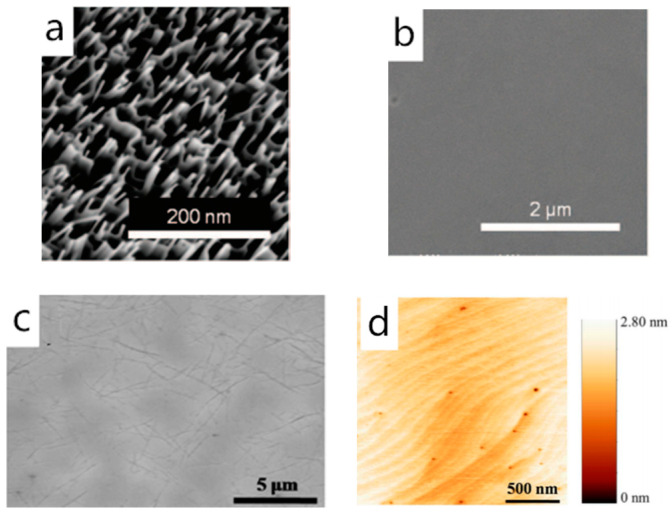
(**a**) SEM image of ZnO nanowalls grown on plasma-treated graphene layers. (**b**) SEM image of GaN thin film grown on ZnO nanowalls on plasma-treated graphene layers; Copyright 2010, The American Association for the Advancement of Science (AAAS). (**c**) SEM image of carbon nanotube–graphene hybrid structure (CGH) on sapphire. (**d**) AFM images of the surface of GaN epilayers grown on CGH. Copyright 2015, Springer Nature [9,48].

From the data listed in Table 2, the adsorption energy of III-nitrides on graphene is higher than the III-arsenides, so theoretically, high-quality III-nitride van der Waals epilayer can be obtained with ease, which is also in line with the experimental results. In 2014, Alaskar et al. pointed out that ideal GaAs van der Waals epilayer was hard to prepare on graphene-covered silicon substrate despite the introduction of Ga prelayer [46]. The E_b_/E_c_ ratio of GaN is as high as 2.77, compared to GaAs, 0.41, and many experiments have demonstrated that qualified GaN film can be prepared through the van der Waals epitaxy method. In 2012, Choi et al. achieved GaN epilayer fabrication on a graphene-coated sapphire substrate without the participation of a low-temperature buffer layer [40]. They found out that continuous GaN film can be obtained on the 0.6 nm-thick graphene, and when the thickness of graphene reached 7.9 nm (more precisely, a thin layer of graphite), the GaN film will become mirror smooth. This implies that during the III-nitride van der Waals epitaxy process, graphene can take the place of traditional low-temperature buffer layer if it is thick enough, which may effectively simplify III-nitrides preparation process. In 2010, Chung et al. demonstrated GaN epigrowth with the interaction of graphene with ZnO nanowalls. Oxygen plasma-treated graphene can provide enough nucleation sites for III-nitrides growth, but it cannot optimize the morphology of the obtained film, that is why the ZnO nanowalls are introduced [9] (Figure 1a,b). Subsequently, in 2016, Chung et al. still used oxygen-treated graphene combined with ZnO nanowalls to generated GaN growth. The surface morphology and diameter of as-grown GaN dots are with high consistency. It should be mentioned that in this experiment, the graphene was layered on the amorphous substrate and patterned into graphene dots with a diameter of 3.5 μm and space of 10 μm [12]. However, the introduction of ZnO nanowalls may result in resistance increasing at the interface. Therefore, in 2015, Seo et al. chose the carbon nanotube-graphene hybrid (CGH) structure to solve the nucleation problem as well as the morphology problem. The experiment result confirmed that the carbon nanotube-graphene hybrid structure had good electrical and thermal conductivity, and the GaN film grown on it was continuous and flat without cracks appearing [48] (Figure 1c,d).

**Figure 2:**
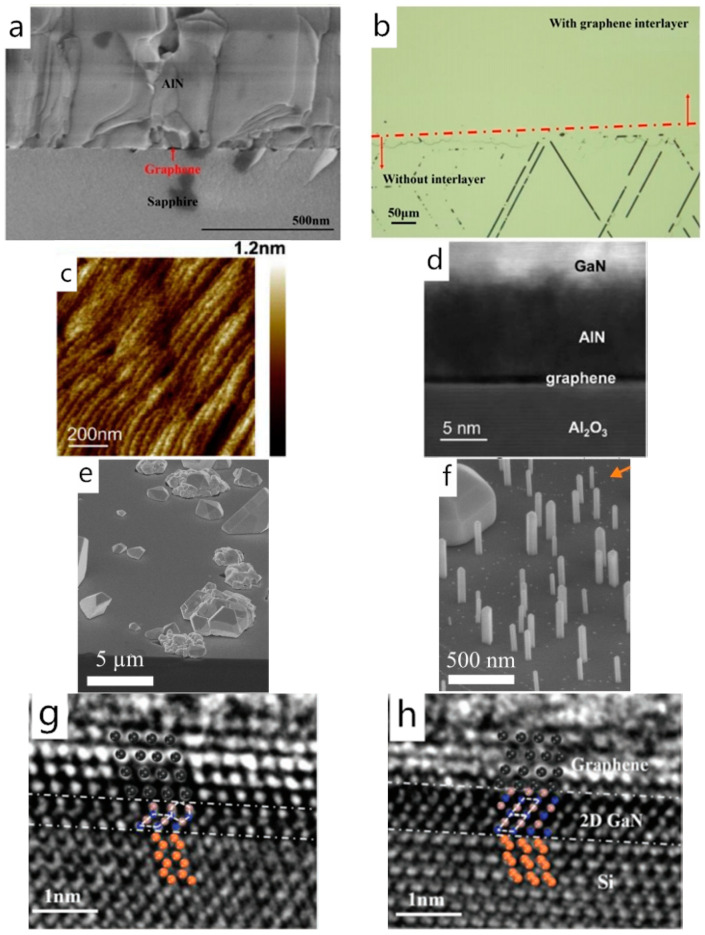
(**a**) Cross-sectional SEM images of AlN layer grown on multilayer graphene-sapphire (G–S). (**b**) Optical microscopy surface section image of GaN grown on AlN–sapphire template (the red dashed line separates the areas with and without multilayer graphene interlayer); Copyright (2017) The Japan Society of Applied Physics. (**c**) Surface morphology of GaN film grown on AlN/multilayer graphene (MLG)/sapphire. (**d**) Cross-sectional STEM image of GaN films grown on AlN/MLG/sapphire; Copyright 2020 Elsevier B.V. All rights reserved. (**e**) SEM image of GaN (nanorods) NRs grown on AlGaN nucleation islands on Si (111) without graphene where meltback etching is observed. (**f**) SEM images of GaN NRs grown on AlGaN nucleation islands on graphene-covered Si (111). Copyright 2016, American Chemical Society. Cross-sectional aberration-corrected TEM images for GaN grown with hydrogenation (**g**) without and (**h**) with the assistance of plasma. Copyright 2019, WILEY-VCH Verlag GmbH & Co. KGaA, Weinheim [13,41,50,51].

In addition to the ZnO and carbon nanotubes, AlN can also act as a prelayer for the GaN epitaxy for its relatively high E_b_/E_c_ ratio and approximate lattice constant with GaN (a_AlN_ = 0.3111 nm, a_GaN_ = 0.3189 nm). Li et al. grew an AlN prelayer on the graphene-coated sapphire substrate as a buffer layer for the subsequent GaN epigrowth in 2017. The test result of the film indicated that the dislocation density in the AlN film showed a significant decline and the GaN film remained crack-free due to the sufficient stress relaxation (Figure 2a,b), which is different from that on the bare sapphire substrate [41]. Besides, the result of DFT calculation demonstrates that Al atoms have higher adsorption energy (1.7 eV) and lower migration barrier (0.03 eV) on graphene surface than that of Ga atoms (1.5 and 0.05 eV, respectively) [47], namely, Al atoms are more feasible to adsorb on the graphene surface and have larger diffusion length than Ga atoms. Therefore, the participation of Al atoms is beneficial for increasing nucleation density and for uniform nucleation distribution. Recently, Zhou et al. pointed out that the GaN film grown on multilayer graphene with a sputtered AlN buffer had a flat morphology, and atomic-step terraces of GaN were visible in the AFM image (Figure 2c,d), which indicated that a 2D growth had happened here. They also performed the GaN growth process without an AlN buffer layer. However, many isolated GaN rods were observed instead of a continuous GaN film, which may result from the insufficient nucleation sites and low adsorption energy of Ga atoms on graphene [50]. In addition to the increased nucleation density, the proper introduction of the Al component in the GaN epitaxy process can also optimize the orientation alignment. Heilmann et al. performed GaN nanorods growth on a graphene-coated silicon substrate in 2016. They claimed that the introduction of the graphene buffer layer solved the lattice symmetry inconsistency and melt-back etching problem, whereas applying AlGaN for nucleation solved the GaN uneven nucleation distribution and random orientation problem [13] (Figure 2e,f).

It should be mentioned that graphene can also act as an encapsulation layer for 2D GaN growth in addition to being a buffer layer for 3D or 1D GaN epitaxy [25,52]. Specifically, 2D GaN has great potential for the development of deep ultraviolet light-emitting diodes, energy conversion devices, etc. for its lattice structure and bandgap can be well controlled. As confirmed by Wang et al., through theoretical calculation and experiment, the lattice structures of 2D GaN would change from R3m structure to P6_3_MC structure when the number of 2D GaN layers changed from six to four (Figure 2g,h), and its band gap would change from 4.18 to 4.65 eV accordingly [51].

**Figure 3:**
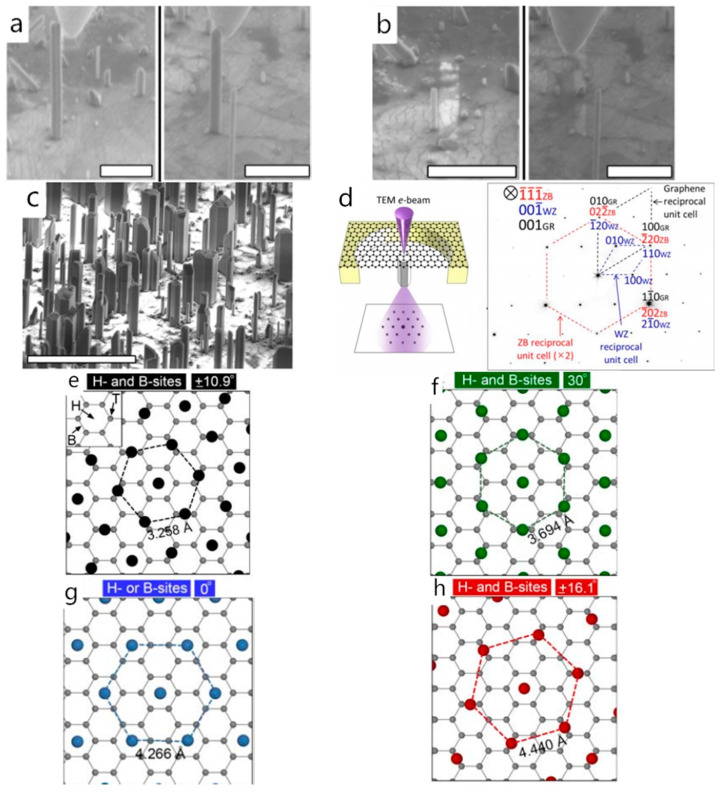
(**a**) SEM image of two different NRs on graphene before bending (scale bar: 2 μm). (**b**) SEM image of two different NRs on graphene after the breaking (scale bar: 2 μm). (**c**) SEM image of GaN micro- and nanostructures grown on the graphene/sapphire substrate (scale bar: 10 μm); Copyright 2015, American Chemical Society. (**d**) Schematic depicting the plan-view TEM observation (left). selected area electron diffraction (SAED) patterns of InAs/S-SLG (right). Copyright 2013, John Wiley and Sons. Relative orientation and arrangement when semiconductor atoms are adsorbed on (**e**,**f**,**h**) H- and B-sites. (**g**) H- or B-sites. Copyright 2012, American Chemical Society [26,35,60].

After discussing the nucleation-growth process of the epilayer, we will focus on its orientation. As for the traditional heteroepitaxy, the epilayer interacts with the substrate through chemical bonds, as a result, the epilayer’s orientation is consistent with the substrate’s orientation. However, the orientation of the van der Waals epilayer is not exactly controlled by the substrate, because the 2D buffer layer can partly screen the potential of the substrate. As a commonly used buffer layer, graphene’s hexagonal lattice structure suits the III-nitrides’ wurtzite or sphalerite structure well. Many studies have demonstrated that the van der Waals epilayer’s orientation can be affected by both the graphene and the substrate. More specifically, the number of graphene layers and the substrate polarity both have an impact on the epilayer’s orientation. [52,53]. In 2012, Choi et al. claimed that a GaN film grown on a graphene-covered sapphire substrate remained the same lattice symmetry and orientation with the underlying sapphire substrate. This may be because that the graphene Choi et al. used was directly grown on the sapphire substrate with a Ni prelayer (the Ni layer was removed after the graphene growth). The as-grown graphene was already impacted by the sapphire substrate and it accordingly affected the orientation of the upper GaN film [40]. The research published by Kim et al. in 2014 also demonstrated that a well-aligned single-crystalline GaN film can be epigrown on a graphene-coated SiC substrate [54]. The graphene used here was prepared on the SiC substrate via Si sublimation, which is known to retain its unique orientation throughout an entire SiC substrate [55,56] and subsequently affect the orientation of the GaN epilayer. However, the impact of transferred graphene on the orientation of the epilayer is related to the number of graphene layers. Graphene is a non-polar 2D material, whose screening effect of the substrate potential is limited but will enhance with the increasing number of layers [57,58]. A DFT calculation was performed by Wei Kong et al. in 2018 to evaluate the penetration distance of the electrostatic potential from substrates and its relationship with the ionicity of substrates. They found that as the polarity of the interatomic bonds of the substrate increased, more layers of graphene were needed to isolate the effect of the substrate for epitaxial growth, and a strong ionicity in 3D materials allowed for the transmission of atomic potential fluctuation beyond the three monolayer graphene distance. The result fitted well with their experiment [59]. Heilmann et al. attempted to investigate the influence of the substrate on the epitaxial orientation in 2015. They first grew GaN nanorods on single-layer graphene covered sapphire substrate and then broke them. Heilmann et al. found that after removing the as-grown GaN nanorods, a small GaN spot remained in some of them, whereas the others left no traces behind (Figure 3a,b). The different situation corresponds to different nucleation-growth conditions, i.e., growing on the sapphire substrate through the nanoholes of graphene and directly growing on the graphene, respectively. Considering that all the as-grown GaN nanorods were well aligned (Figure 3c), it can confirm that the underlying substrate potential can indeed penetrate the graphene layer and play a role in controlling the orientation of the epilayer [35]. The study published by Zeng et al. in the following year also confirmed this view. They prepared AlN van der Waals epilayer on a bilayer graphene-coated sapphire substrate. The selected area electron diffraction (SAED) pattern of the AlN domain showed a single diffraction pattern of the (0001) c-axis-oriented wurtzite structure. The orientation relationships were (0001) _AlN_|| (0001) _sapphire_, [1100] _AlN_|| [1210]_sapphire_, which was consistent with the epitaxial relationship of AlN film grown on the bare sapphire substrate [10]. However, if the number of graphene layers continues to increase, the orientation of the epilayer will be mainly controlled by the graphene, not the substrate below [3]. As Alaskar et al. confirmed that during the GaAs van der Waals epitaxy process on multilayer graphene covered silicon substrate, the orientation of the epilayer is controlled by the underlying graphene layer despite the poor crystallinity [47]. A generic model proposed by Munshi et al. can explain possible structures of semiconductor/graphene interfaces and different atomic arrangements of the semiconductor atoms on graphene [60,61]. They pointed out that the (0001) plane of a hexagonal semiconductor ((111) plane of a cubic semiconductor) is energetically preferred to nucleate on graphene due to the lattice symmetry, which subsequently determines the out-of-plane epitaxial relationship for epilayer. However, the in-plane orientation depended on which sites the semiconductor atoms adsorbed on. There are three possible adsorption sites for semiconductor atoms on the surface of graphene: (1) above the center of hexagonal carbon rings of graphene (H-site), (2) above the bridge between the carbon atoms (B-site), and (3) above the top of a carbon atom (T-site), shown in the inset of Figure 3e. The T-site is an unfavorable adsorption site for semiconductor atoms due to its lower adsorption energy and will not be discussed here. For different types of semiconductors and preferential adsorption sites, the atomic arrangements were various when atoms were adsorbed: (1) on both H- and B-sites (Figure 3e,f,h) and (2) on either H- or B-sites (Figure 3g), which is to say that the preferred atomic arrangements guided the in-plane orientation of epilayer.

**Figure 4:**
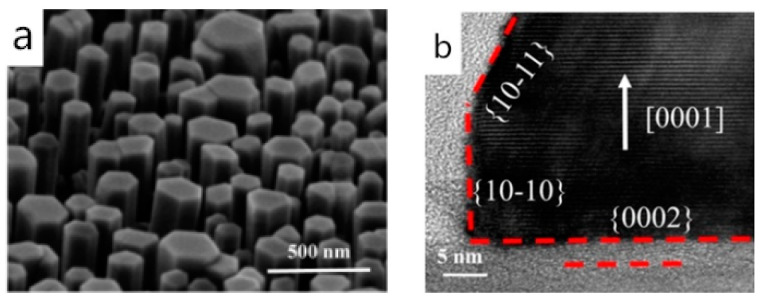
(**a**) Tilted view SEM images of AlGaN nanowires grown on graphene/SiO_2_/Si (100) substrate at a temperature of 1090 °C. (**b**) Cross-sectional TEM image of the nanowire. Copyright 2019, American Chemical Society [42].

Compared to the graphene-covered crystalline substrate structure, the graphene covered amorphous substrate structure, as well as the suspended graphene, can further prove that graphene can certainly affect the orientation of the epilayer. Hong et al. grew the InAs double-heterostructure on suspended graphene in 2013. The SAED pattern taken at the InAs/graphene/InAs interface showed that the InAs nanostructure grew along the [111] direction, and the epitaxial orientation relationship was ($\bar{1}\bar{1}\bar{1}$)[211] _InAs_‖(001)[100] _SLG_ (Figure 3d). They pointed out that the InAs nanostructure on both sides of the suspended graphene was about 0.62 nm apart, which exceeded the van der Waals attraction distance [26]. Therefore, it can be concluded that the orientation of the as-grown InAs nanostructure was affected by the graphene. Subsequently, in 2019, Wang et al. performed AlGaN nanowire growth on the graphene-covered amorphous substrate and achieved hexagonal AlGaN nanowire growth with a flat top by adjusting parameters such as pressure, temperature, and V/III ratio (Figure 4a). The obtained single-crystalline AlGaN nanowires grown along <0001> direction indicated that the graphene indeed can regulate the epilayer’s orientation [42,62] (Figure 4b).

In this section, we discussed the nucleation-growth process and the orientation relationship of III-nitrides van der Waals epitaxy growth. Factors such as graphene quality, wettability between the epilayer and the graphene, etc. may restrict the utilization of van der Waals epitaxy for III-nitride-based optoelectronic devices. However, for the compounds with a high E_b_/E_c_ ratio on the graphene, such as GaN and AlN, there are already a considerable amount of studies that have confirmed that the performance of GaN-based and AlN-based LEDs prepared by van der Waals epitaxy is acceptable. The following section will focus on the practical applications of van der Waals epitaxy for III-nitride-based LED fabrication.

## 4. Practical Application of III-Nitrides van der Waals Epitaxy

For traditional heteroepitaxy, a high-quality epilayer can only be obtained when the lattice and thermal mismatch between the epilayer and the substrate is negligible. This puts restrictions on the choice of substrates. Generally, a SiC or a sapphire substrate is required for III-nitrides growth, as well as a tedious low-temperature buffer layer. Besides, the strong chemical interaction between the epilayer and the substrate limits the exfoliation and transfer of the epitaxy structure and the reuse of the substrate. Thus, van der Waals epitaxy is raised trying to alleviate the problems mentioned above.

**Figure 5:**
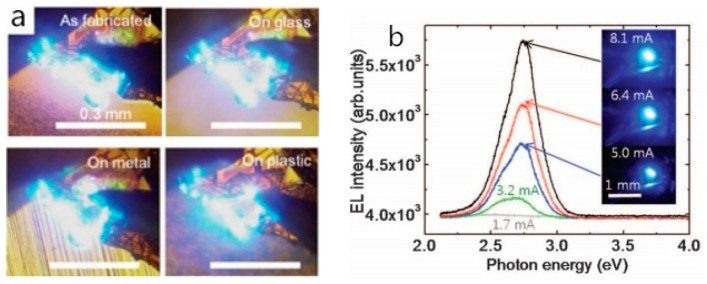
(**a**) Optical images of light emissions from the as-fabricated light-emitting diode (LED) on the original substrate and transferred LEDs on the foreign metal, glass, and plastic substrates. (**b**) Room-temperature electroluminescence (EL) spectra of the LED transferred onto a plastic substrate. Optical microscopy images show the light emission at different applied current levels of 1.7–8.0 mA. Copyright 2010, The American Association for the Advancement of Science (AAAS) [9].

In recent years, many researchers committed to qualified III-nitride van der Waals epilayer preparation and realized high-performance III-nitride-based LED fabrication with the assistance of graphene. In 2010, Chung et al. demonstrated that the GaN-based blue LED can be successfully prepared on the graphene-coated sapphire substrate through the van der Waals epitaxy method. The experimental result indicated that the as-fabricated LED can be easily transferred to metal, glass, and plastic substrates and can still emit high-brightness blue light after the transfer process (Figure 5a,b). However, the devices’ resistance increased due to increased interface resistance [9]. This research provides a new pathway for high-power, large-scale, or flexible GaN-based LED fabrication. Kim et al. performed a LED structure epitaxy on a recycled graphene/SiC substrate in 2014 [54]. The test result confirmed that GaN crystal lattice was well aligned at the GaN/graphene/SiC interface, and it was comparable with that of GaN grown on a fresh graphene/SiC substrate though the substrate has been previously used three times. After finishing the LED fabrication, Kim et al. released the whole LED structure at the GaN/graphene interface and found that the as-fabricated LED had a good rectification characteristic and can emit visible blue light.

The introduction of graphene can also enhance the light extraction of LEDs. In 2017, Li et al. grew the n-GaN/5-period In_0.2_Ga_0.8_N/GaN MQWs/Al_0.2_Ga_0.8_N EBL/p-GaN LED structure on the graphene-coated sapphire substrate and tested its performance. Compared to the LED fabricated by the traditional method under the same growth parameters, the as-fabricated LED showed a decreasing turn-on voltage with a good rectification behavior. Both light output power (LOP) and external quantum efficiency (EQE) of it were increased. The LED’s EQE is the product of internal quantum efficiency (IQE) and light extraction efficiency (LEE). Considering that the as-fabricated device’s IQE was decreased due to the poor crystalline quality of the as-grown GaN film, the LEE played an important role in the significant EQE improvement. It may attribute to the multilayer graphene interlayer. In detail, the high refractive index of graphene and the holes in the as-grown GaN epilayer can both contribute to light scattering, i.e., the direction of the propagation of light may change from being regular to being random and have more opportunities to be extracted to the outside, leading to the enhanced light extraction [41]. 

**Figure 6:**
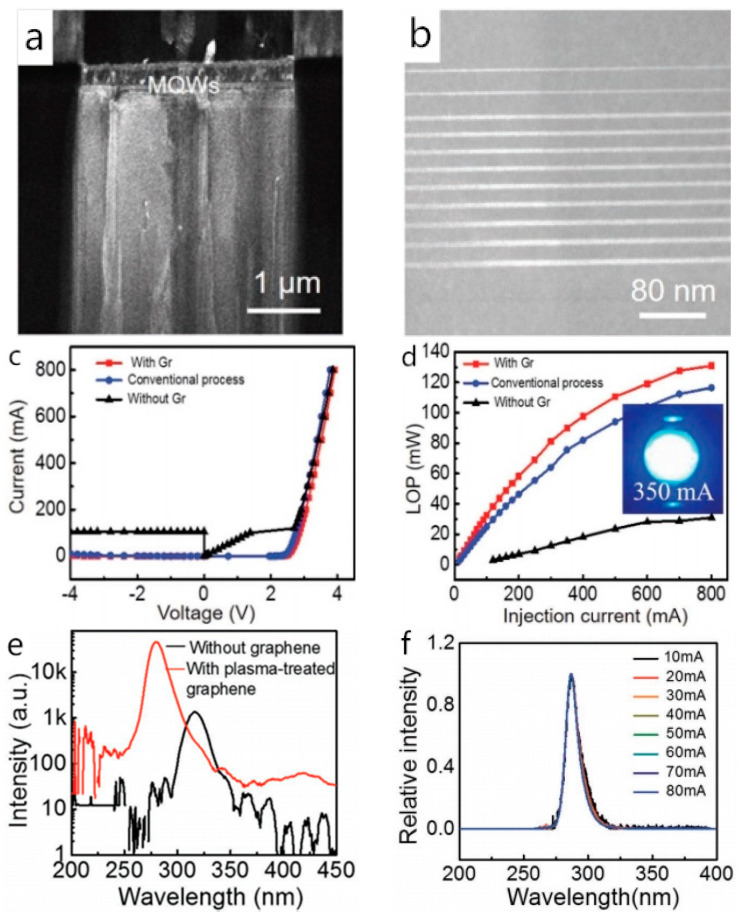
(**a**) Darkfield image of heterostructure with g = 0002. (**b**) Cross-sectional STEM image of In_x_Ga_1−x_N/GaN (multiple quantum wells) MQWs in the as-fabricated blue LED. (**c**) Current-voltage characteristics of the as-fabricated LEDs with and without graphene (Gr) interlayer, and conventional process derived one. (**d**) Light output power of the as-fabricated LEDs with and without Gr interlayer, and conventional process derived one as a function of injection current; Copyright 2018, John Wiley and Sons. (**e**) Electroluminescence (EL) spectra of the (deep ultra-violet) DUV-LEDs with and without graphene interlayer. (**f**) The normalized EL spectra of DUV-LEDs on graphene/sapphire with currents ranging from 10 to 80 mA. Copyright 2019, John Wiley and Sons [14,28].

With a better understanding of the van der Waals epitaxy, the performance of III-nitride-based LEDs prepared using the van der Waals epitaxy method is optimized. In 2018, Chen et al. successfully prepared a high-brightness blue LED directly on a graphene-coated substrate and characterized its structure and electrical properties. The as-grown MQWs presented an abrupt interface and uniform In doping (Figure 6a,b). The I–V curve showed that the as-fabricated LED had a turn-on voltage of 2.5 V and a leakage current of 2 mA at −4 V. It is worth mentioning that the light output power of the as-fabricated LED is 19.1% higher than the traditional ones without a graphene interlayer at the injection current of 350 mA due to sufficient stress relaxation [28] (Figure 6c,d). Subsequently in 2019, Chen et al. prepared a high-quality AlN film on a graphene-coated sapphire substrate and carried out the epigrowth of 20-period AlN/AlGaN SLs/n-Al_0.55_Ga_0.45_N/Al_0.4_Ga_0.6_N/Al_0.5_Ga_0.5_N MQWs/p-AlGaN structure. After the characterization, they pointed out that the as-fabricated DUV-LED had a smoother and flatter morphology, as well as better electrical properties than that without the graphene interlayer. Compared to the DUV-LED without the graphene interlayer, its dominant EL peak was located at 280 nm without shifting despite the changing current, and the EL intensity was increased by two orders of magnitude owing to the negligible residual stress [14] (Figure 6e,f).

**Figure 7:**
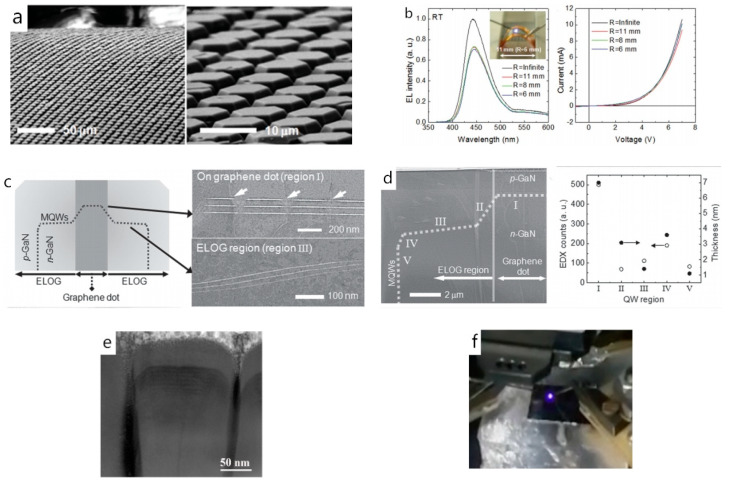
(**a**) Low-magnification (left) and high-magnification (right) FE-SEM images of GaN micro-LEDs under bending radii of 0.6–0.8 mm. (**b**) Room temperature EL spectra (left) and I–V curves (right) of the micro-LEDs under various bending radii. The inset in (b) shows a light emission image of the micro-LEDs at a bending radius of 6 mm. (**c**) Schematic illustration of the micro-LED. STEM images of MQWs formed in the graphene dot region (top image) and ELOG region (bottom image). (**d**) Cross-sectional STEM image of a GaN micro-LED. EDX intensities of the indium content and (quantum well) QW thickness at various QW regions; Copyright 2016, John Wiley and Sons. (**e**) Cross-sectional STEM image of a single AlGaN nanorod LED. (**f**) Optical image of the violet electroluminescence from the AlGaN nanorod LEDs. Copyright 2018, MDPI [12,64].

Additionally, van der Waals epitaxy fits well with the III-nitride-based flexible device fabrication. Flexible substrates usually cannot tolerate the high temperature required for III-nitrides MOCVD growth; the easy-transfer characteristic of van der Waals epitaxy may provide a new pathway for III-nitride-based devices fabrication despite the thermal budget restrictions. [53] Early in 2011, Lee et al. prepared a GaN/ZnO core-shell heterostructure (i.e., n-GaN, InGaN/GaN MQWs, and p-GaN layers coated coaxially around the ZnO nanorod) on a graphene-coated amorphous substrate through van der Waals epitaxy and realized full functional flexible LED fabrication. The as-fabricated nanorods LEDs were transferred to a Cu-coated PET substrate and the test result showed that its luminescence was not degraded after bending. The as-fabricated flexible LEDs can emit blue light under the injection current of 10 mA at bending radii of ∞, 5.5, and 3.9 nm [63]. In 2014, Chung et al. fabricated well-aligned GaN-based microrod LEDs on a graphene-coated SiO_2_/Si substrate. After the transfer process, the microrod LEDs showed great flexibility and its optical performance did not change despite over 1000 bending cycles [38]. Subsequently, they prepared a GaN/graphene heterostructure on the SiO_2_/Si substrate to fabricate flexible GaN micro-LED arrays in 2016. The micro-LEDs prepared here had a similar structure to the traditional blue LEDs, i.e., the n-GaN/In_x_Ga_1−x_N/GaN MQWs/p-GaN structure. The difference was that the micro-LEDs were fabricated on graphene dot patterns (Figure 7a). The application of this structure imparted mechanical flexibility to the device, as Chung et al. confirmed that the EL peak of the as-fabricated micro-LEDs had almost the same shapes, intensities, and positions regardless of the bending radius over the range of 6–11 mm (Figure 7b). However, it also resulted in uneven thickness and composition in the MQWs, which may be responsible for the observed changes in EL peak positions and FWHM values when the bias voltage increased [12] (Figure 7c,d). In 2018, Ren et al. fabricated and characterized AlGaN-based nanorod LEDs on a graphene-coated silicon substrate. The n-AlGaN/MQWs/p-AlGaN layered structure with the abrupt interface and uniform thickness was visible in the STEM image (Figure 7e). The temperature-dependent PL spectra demonstrated that the IQE of the as-fabricated nanorod LEDs was 2.6%. Although the core-shell structure was not completely formed here, it can still emit visible violet light under the 50 mA injection current (Figure 7f). This research can provide a novel way to flexible GaN-based LEDs fabrication if the crystalline quality and the coalescence situation of the GaN nanorods are optimized [31,64].

Some successful cases of III-nitride-based LEDs prepared through van der Waals epitaxy are introduced in this section. Many advantages possessed by the van der Waals epitaxy, such as effective stress relaxation and transferable property can contribute to high-performance and flexible device fabrication. However, there are still some problems worth exploring, including large-scale single-crystalline epilayer preparation, uniform doping, etc. which are essential to further improve the devices’ performance.

## 5. Summary and Prospect

Van der Waals epitaxy based on 2D materials have attracted much attention in recent years due to its novel properties. This paper mainly focuses on the 2D materials, the III-nitride van der Waals epitaxy process on the 2D material, and the III-nitride-based devices fabricated by van der Waals epitaxy.

Graphene is the first 2D material discovered by the scientific community. The remarkable feature of graphene is that the in-plane C atoms interact with each other through strong covalent bonds while the interaction between adjacent graphene layers is weak van der Waals force. The strong in-plane interaction makes graphene tolerant to the high temperature required for III-nitrides epitaxy, and the weak van der Waals force between the adjacent layers facilitates the exfoliation and transfer of the epilayer. Additionally, the excellent in-plane conductivity and optical transparency of graphene make it act as a transparent conductive electrode without peeling-off, which can simplify the device fabrication procedure. In addition, many studies have demonstrated that completely large-scale graphene can be obtained through the CVD or SiC substrate sublimation method.

Using graphene as a buffer layer or encapsulation layer for the III-nitride van der Waals epitaxy can facilitate the novel III-nitride-based device development. However, there are still some problems left. The free-of-dangling bonds characteristic of the graphene surface is unfavorable for III-nitrides nucleation and may involve 3D island growth mode for those low-adsorption materials, which is unfavorable for flat and smooth film fabrication. Some solutions are proposed to increase the chemical reactivity of the graphene surface, such as plasma treatment and nanostructures growth. However, it may increase the device’s contact resistance at the same time. Therefore, the methods for increasing the chemical reactivity of the graphene surface need further optimization. Besides, the low migration barrier of atoms allows them to migrate rapidly on the graphene surface, which can sufficiently reduce the epitaxy time. However, it also enlarges the problem of unstable atomic adsorption on the graphene surface. Thus, better wettability between the epilayer and the graphene and optimized epitaxy parameters are required for qualified III-nitride epilayer preparation.

As for the III-nitride-based LEDs, the application of van der Waals epitaxy is conducive for simplifying the lift-off procedures, which will help to broaden the application scenarios. The related research work of 2D GaN and van der Waals solids also lights the way for III-nitride-based novel devices fabrication. However, some obstacles, including large-scale single-crystalline epilayer preparation (2-in wafer-scale GaN van der Waals epilayer is available now [65]), uniformity of epilayer doping, and contact resistance reduction of the device still need to overcome.

## Figures and Tables

**Table 1:** Recent examples of III-nitride epilayer preparation through van der Waals epitaxy.

Growth Method	Substrate	2D Material	Epilayer	Reference
MOCVD	Sapphire	Graphene	GaN	[9]
MOCVD	Sapphire	Graphene	AlN	[10]
MOCVD	Sapphire	Graphene	AlN/GaN	[11]
MOCVD	SiO_2_/Si	Graphene	GaN	[12]
MOCVD	Si	Graphene	GaN	[13]
MOCVD	SiC	Graphene	AlN	[14]
MOCVD	SiC	Graphene	GaN	[15]

**Table 2:** E_b_/E_c_ ratio of III–V compound and graphene system [47,49].

Binary Materials	Bulk Cohesive Energy E_c_/(eV)	Adsorption to Bulk Cohesive Energy Ratio E_b_/E_c_
GaAs	6.7	0.41
AlAs	7.7	0.39
GaN	2.2	2.77
AlN	2.9	2.17
